# Conditional Deletion of PDK1 in the Forebrain Causes Neuron Loss and Increased Apoptosis during Cortical Development

**DOI:** 10.3389/fncel.2017.00330

**Published:** 2017-10-20

**Authors:** Congyu Xu, Linjie Yu, Jinxing Hou, Rosemary J. Jackson, He Wang, Chaoli Huang, Tingting Liu, Qihui Wang, Xiaochuan Zou, Richard G. Morris, Tara L. Spires-Jones, Zhongzhou Yang, Zhenyu Yin, Yun Xu, Guiquan Chen

**Affiliations:** ^1^State Key Laboratory of Pharmaceutical Biotechnology, Model Animal Research Center, Ministry of Education (MOE) Key Laboratory of Model Animal for Disease Study, Nanjing University, Nanjing, China; ^2^Department of Neurology, Nanjing Drum Tower Hospital, Nanjing University Medical School, Nanjing, China; ^3^Centre for Cognitive and Neural Systems, University of Edinburgh, Edinburgh, United Kingdom; ^4^Consejo Superior de Investigaciones Científicas, Universidad Miguel Hernández, Instituto de Neurociencias, Alicante, Spain; ^5^Centre for Dementia Prevention, University of Edinburgh, Edinburgh, United Kingdom; ^6^Euan MacDonald Centre for Motor Neurone Disease Research, University of Edinburgh, Edinburgh, United Kingdom; ^7^Department of Geriatrics, Nanjing Drum Tower Hospital, Nanjing University Medical School, Nanjing, China

**Keywords:** PDK1, Akt, mTOR, neuron loss, apoptosis, learning and memory

## Abstract

Decreased expression but increased activity of PDK1 has been observed in neurodegenerative disease. To study *in vivo* function of PDK1 in neuron survival during cortical development, we generate forebrain-specific *PDK1* conditional knockout (cKO) mice. We demonstrate that *PDK1* cKO mice display striking neuron loss and increased apoptosis. We report that *PDK1* cKO mice exhibit deficits on several behavioral tasks. Moreover, *PDK1* cKO mice show decreased activities for Akt and mTOR. These results highlight an essential role of endogenous PDK1 in the maintenance of neuronal survival during cortical development.

## Introduction

PDK1, a kinase in the PI3K signaling pathway, plays pivotal roles in various biological processes such as cell growth, longevity, and metabolism (Engelman et al., 2006). Activated PI3K phosphorylates phosphatidylinositol bisphosphate (PIP_2_) to generate phosphatidylinositol trisphosphate (PIP_3_). PDK1 is recruited to the intracellular membrane by PIP_3_ via its PH domain and then phosphorylates Akt at the 308 threonine (Thr) residue (Alessi et al., 1997; Calleja et al., 2007). On the other hand, the lipid phosphatase PTEN deactivates PDK1 by transforming PIP_3_ to PIP_2_ (Engelman et al., 2006). AGC kinase members such as p90 ribosomal S6 kinase 1/2 (RSK1/2), p70 ribosomal S6 kinase (p70S6K), serum- and glucocorticoid-induced protein kinase (SGK), and protein kinase C (PKC) are substrates for PDK1 (Mora et al., 2004; Kharebava et al., 2008).

Due to embryonic lethal effect caused by PDK1 knockout in the whole body, it has been impossible to use *PDK1^-/-^* mice to study *in vivo* functions of PDK1 in the postnatal cerebral cortex. The advantage of viable cell-type specific *PDK1* conditional knockout (cKO) mice has helped solve this problem. Recent work showed that conditional deletion of PDK1 through *GFAP-Cre* mediated gene recombination causes microcephaly in mice, indicating a critical role of PDK1 in brain development (Chalhoub et al., 2009). A conditional knock-in mouse model expressing the PDK1 L155E mutation displays microcephaly as well (Cordon-Barris et al., 2016). In addition, it has been demonstrated that PDK1 in neural progenitor cells (NPCs) is important for the generation of oligodendrocyte precursor cells (Watatani et al., 2012) and neuronal migration (Itoh et al., 2016). The work on epidermis-specific *PDK1* cKO mice has revealed an essential role of PDK1 in asymmetric cell division (Dainichi et al., 2016).

Recent evidence has shown that PDK1 is involved in neurodegenerative disease, but how it exerts its role is controversial. On one hand, reduced PDK1 levels (Liu et al., 2011) and impaired PI3K signaling (Steen et al., 2005; Talbot et al., 2012) were found in Alzheimer’s disease (AD) brain, suggesting a loss-of-function manner. On the other hand, another group reported that PDK1 activity was increased in AD brain, and that PDK1 inhibitor enhanced α-secretase activity and reduced amyloid plaques in APP transgenic (Tg) mouse models of AD (Pietri et al., 2013), suggesting a gain-of-function mechanism.

Kharebava et al. (2008) have recently demonstrated that over-expression of PDK1 reduces trophic deprivation (TD)-induced apoptosis, and that RSK1/2 is required for PDK1-mediated neuroprotection. However, it remained uninvestigated whether endogenous PDK1 plays a critical role in neuronal survival during cortical development. We aimed to address this question in the present study. Here, we have crossed *PDK1^f/f^* mice with a forebrain neuron-specific *Cre* Tg line (Gorski et al., 2002) to generate *PDK1* cKO (*PDK1^f/f^;Emx1-Cre*) animals, in which PDK1 is inactivated in excitatory neurons of the cortex. We show that PDK1 levels were significantly reduced in the cortex of *PDK1* mutant mice. We report that *PDK1* cKO mice display striking neuron loss, abnormal apoptosis, and severe memory deficit. We find that *PDK1* cKO mice exhibit decreased Akt/mTOR activities. These findings highlight a protective role of endogenous PDK1 during brain development.

## Materials and Methods

### Animal Care and Use

*PDK1* cKO mice were generated by crossing floxed *PDK1^f/f^* mice (Feng et al., 2010) with *Emx1-Cre* Tg (Gorski et al., 2002). *Emx1-Cre* and *mTmG* mice (Muzumdar et al., 2007) were purchased from the Jackson Laboratory (Bar Harbor, ME, United States). To generate *PDK1* cKO mice, we crossed homozygous *PDK1^f/f^* with *Emx1-Cre* to obtain *PDK1^f/+^;Emx1-Cre* mice, which were then bred with *PDK1^f/f^* to get *PDK1^f/f^;Emx1-Cre* (*PDK1* cKO). *PDK1^f/+^;Emx1-Cre* mice grow normally and their brain morphology did not differ from that of *PDK1^f/+^* or *PDK1^f/f^* mice in our analyses. Therefore, *PDK1^f/+^;Emx1-Cre* and *PDK1^f/f^* served as littermate controls to *PDK1* cKOs. To detect the floxed *PDK1* allele, the following primers were used. The forward primer is TGTGCTTGGTGGATATTGAT and the reverse primer is AAGGAGGAGAGGAGGAATGT.

The mice were kept on 7:00AM–19:00PM light cycle under conditions of constant humidity and temperature (25 ± 1°C). The mice were group-housed (4–5 per cage) throughout the experimental period and had *ad libitum* access to food and water. Both male and female mice were used in this study. Behavioral experiments were conducted during the light phase of the cycle (8:00AM–18:00PM). Different cohorts of mice were used for behavioral and biochemical experiments. The mice were bred and maintained in an SPF level of animal room in the core facility of the Model Animal Research Center (MARC) at Nanjing University. The genetic background of the mice used in this study was C57BL/6. Mouse breeding was conducted under IACUC approved protocols at the MARC. All the experiments were performed in accordance with the Guide for the Care and Use of Laboratory Animals of the MARC at Nanjing University.

### Brain Lysate Preparation

Mice were euthanized by CO_2_ at 3 weeks or 3 months of age. Tissues from various brain areas were quickly collected and then placed into liquid nitrogen. Cortical samples were homogenized in cold RIPA (radio immunoprecipitation assay) lysis buffer [consisting of the following (in mM): 20 mM Tris–HCl, pH 7.4, 150 mM NaCl, 1 mM EDTA, 1% NP-40, 0.5% sodium deoxycholate, and 0.1% SDS] containing protease and phosphatase inhibitors (Thermo). Lysates were cleared by centrifugation (14,000 rpm for 15 min). Samples were stored at -80°C until use. Protein concentration was analyzed using a standard BSA method.

### Western Blotting

Normalized cortical samples (40 μg total protein) were used in 12% SDS–PAGE (Invitrogen) and then transferred to nitrocellulose membrane. After blocking with 5% (w/v) dry milk for 1 h, membranes were incubated with primary antibodies overnight and reacted with infrared dye-coupled secondary antibodies (goat anti-rabbit IRdye800, goat anti-rabbit IRdye680, goat anti-mouse IRdye800, or goat anti-mouse IRdye680). Membranes were scanned using the Odyssey Infrared Imaging System (Li-Cor). The following antibodies were purchased from the CST: total-Akt (1:1000), pAkt^S473^ (1:600), GSK3α/3β (1:1000), pGSK3α^S21^/3β^S9^ (1:600), pAkt^T308^ (1:500), pAkt substrates (1:500), pPKA substrates (1:500), pCREB^Ser133^ (1:400), pS6K^Thr389^ (1:1000), S6K (1:1000), pS6^Ser235/236^ (1:1000), and S6 (1:1000). The following antibodies were also used: anti-PDK1 (1:1000, Thermo), PKA Reg1α (1:1000, Santa Cruz), NeuN (1:500, Millipore), β-actin (1:10000, Sigma–Aldrich), and GAPDH (1:10000, Genetex).

### Paraffin Brain Blocks and Nissl Staining

The mouse at 3 weeks or 3 months was placed into a CO_2_ box for about 10 s so that its respiration became quite faint and its heartbeat got weak. Immediately after the CO_2_ treatment, cardiac perfusion was conducted using 15 ml 4% paraformaldehyde (PFA) solution (in phosphate buffer saline, PBS) in a well-ventilated hood. The brain was dissected out and then fixed in PFA overnight. After fixation, the brain was dehydrated and embedded in paraffin.

After embedding, several paraffin brain blocks were prepared. In each block, 4 hemi-brains, including 2 controls and 2 cKOs, were placed together and were sectioned sagittally (10 μm) using a microtome (Leica Microsystems, Bannockburn, IL, United States). This way allowed 4 brain sections (2 from the control and 2 from the cKO group), which were on identical stereotaxic plane, to be placed on the same slide. For embryos, the head was fixed in 4% PFA overnight and was later embedded in paraffin. Coronal brain sections for each embryo were collected individually.

Sections were incubated at 58°C for 1 h, deparaffinized in xylene and re-hydrated. They were rinsed in PBS for 5 min, soaked in 0.5% cresyl violet for 12 min, and then dehydrated by a series of ethanol (70, 90, 95, and 100%). After the sections were cleared in xylene, they were coverslipped with neutral resin (Sinopharm Chemical Reagent Co., Ltd., Shanghai).

### The Cortex Volume, Neuron Counting, and Cell Density

A method described by us previously (Tabuchi et al., 2009; Chen et al., 2010) was used to measure the cortex volume and to count the total number of neurons. It was based on an unbiased stereological neuron counting technique (West and Gundersen, 1990).

The following experiments were conducted to measure the cortex volume for each hemi-brain. First, for each paraffin block, a total of 8 brain slides spaced 400 μm apart were selected for Nissl staining. Since each paraffin block contained 2 control and 2 cKO hemi-brains, there were 4 brain sections on each slide. The section thickness was 10 μm. Second, Nissl-stained images were captured and the area for the cortex in each brain section was measured using the Olympus CellSens Standard system. For each mouse, areas for the cortex from 8 (control) or 6 (cKO) sections were averaged to obtain the mean value across sections. Third, the volume of a hemi-cortex was calculated using the following formula: volume = the mean area × the total thickness of a hemi-brain. The latter was 3200 μm for control but 2400 μm for cKO.

The following experiments were performed to calculate the total number of cortical NeuN+ cells for each hemi-brain. First, NeuN immunohistochemistry (IHC) was conducted using 8 slides spaced 400 μm apart from each paraffin brain block. Second, for each NeuN-stained section, a total of 10 microscopic fields were randomly selected under the 40× magnification lens of an Olympus BX53 microscope. Each microscopic field was 100 μm × 100 μm × 10 μm (10^5^ μm^3^) in size and was defined as a counting unit. Third, the total number of NeuN+ cells in each counting unit was counted. The numbers were then averaged across sections to obtain a mean value for NeuN+ cell number per unit. The cell density was defined as the number of NeuN+ cells in a 1 mm^3^ area. It was calculated using the following formula: density = mean number of NeuN+ cell/mm^3^. Fourth, the total number of cortical NeuN+ cells in each hemi-brain was calculated using the following formula: total number = cell density × the cortex volume.

For the measurement of the diameter of cell bodies of NeuN+ cells, a total of 345 cells in the NeuN-stained images for *PDK1* cKO at 3 weeks and 365 cells in *PDK1* cKO at 3 months were randomly selected to calculate the averaged value (*n* = 3 mice/group/age).

### BrdU Labeling

BrdU (B5002, Sigma–Aldrich) was administered to pregnant dams at the concentration of 100 mg/kg. To label proliferating NPCs, BrdU was intraperitoneally injected to pregnant dams at E13.5, E15.5, and E17.5. Embryonic brains were collected 30 min after the injection and were then processed for paraffin embedding. Each paraffin block contained only one embryonic brain, and serial coronal sections were prepared using a microtome.

### Immunohistochemistry and Cell Counting

Brain sections were immunostained with antibodies against NeuN (1:500, Millipore), PDK1 (1:1000, Abcam), SVP38 (1:500, Millipore), microtubule-associated protein 2 (MAP2) (1:500, Millipore), BrdU (1:500, Abcam), PH3 (1:500, CST), GFAP (1:500, Sigma–Aldrich), and Iba1 (1:500, Wako). For fluorescence IHC (FIHC), sections were incubated with Alexa Fluor 488 secondary antibodies (Invitrogen, 1:500) or Cy3/Cy5 secondary antibodies (Jackson ImmunoResearch; 1:500). Immunofluorescence images were captured using a Leica TCS SP5 laser confocal microscope except for MAP2 staining.

For BrdU+ cell counting, three coronal sections spaced 200 μm apart from each embryo were used. Images for cortical BrdU staining were captured under the 40× objective lens of a Leica confocal laser scanning microscope. In each image, 2 counting units were randomly selected and each unit was an area of 100 μm (surface of the ventricular zone, VZ) × 200 μm (vertical to the VZ surface). The total number of BrdU+ cells in each unit was counted. For each embryo, the number of BrdU+ cells was averaged across 6 counting units to make the mean number.

For PH3+ cell counting, three coronal sections spaced 200 μm apart from each embryo were used. Images were captured under the 10× objective lens of a Leica confocal laser scanning microscope. The total number of PH3+ cells on the surface of VZ was counted. The number was then averaged across 3 sections to obtain the mean number of PH3+ cells for each embryo.

### TUNEL Staining

Brain sections were blocked with 5% goat serum for 30 min and then treated with the TUNEL (terminal deoxynucleotidyl transferase-mediated dUTP-biotin nick end-labeling) BrightGreen Apoptosis Detection Kit (Vazyme) at 37°C for 1 h (Tabuchi et al., 2009). The sections were washed using TBS (Tris-buffered saline) for three times and then scanned using a Leica confocal laser scanning microscope.

TUNEL was also co-stained with NeuN (1:1000, Millipore), Tuj1 (1:1000, SAB), and GFAP (1:500, Sigma–Aldrich). Sections were incubated with Alexa Fluor 488 secondary antibodies (Invitrogen, 1:500) or Cy3/Cy5 secondary antibodies (Jackson ImmunoResearch; 1:500). Images were captured using a ZEISS LSM 880 confocal laser scanning microscope.

### Analysis of Dendritic Length

Dendrites for neurons in cortical layer V and hippocampal CA1 were analyzed. Ctip2 antibody was used to label pyramidal neurons in the above brain regions. Double immunostaining on Ctip2/MAP2 was conducted using brain sections at 3 weeks and 3 months. After the FIHC experiments, images doubly positive for Ctip2/MAP2 were examined and observed under the 20× objective lens of a ZEISS LSM 880 confocal laser scanning microscope. By this way, we could identify Ctip2+ cells displaying long MAP2+ dendrites in cortical layer V. For each mouse, we examined 2–3 brain sections to trace 10 cortical Ctip2+ cells which sent long and unbroken MAP2+ dendrites to cortical layer I/II. In hippocampal CA1, apical dendrites of Ctip2+ cells were quite long and it was relatively easy to get measured. Images were captured under the 10× objective lens and then processed by ImageJ^1^ to measure dendritic length. For each brain area, a total of 30 Ctip2+ neurons from three mice per group were analyzed.

### Morris Water Maze Test

The water maze is a circular pool (1.6 m in diameter). In the hidden platform task, the platform (10 cm in diameter) was kept under water and maintained in the same position. The mice were trained to learn the hidden platform with four trials per day (the inter trial interval, ITI = 15 min) for 5 days. Each training trial lasted for 60 s (s). If the mice were unable to find the platform, they were guided to it by hand and were allowed to stay on it for 30 s. The swimming path of the mice was monitored using the ANY-Maze^®^ tracking system (ANY-Maze, Stoelting Co., Wood Dale, IL, United States). After the last training trial of 24 h on day 5, the mice were subjected to a probe test in which the platform was removed and the mice were allowed to search for it for 60 s.

### Rotarod Test

The rotarod test was conducted in the same experimental room for the open-field test. The mice were placed in a neutral position on a stationary rotarod (3 cm in diameter, ShangHai Biowill Co., Ltd., Shanghai). Timers were used to record the time to fall. Mice were tested on the rotarod at constant rotation speeds of 10, 20, 30, and 40 rpm/min.

### Open-Field Test

The ANY-Maze^®^ system was used to monitor locomotion of the mice. A 40 cm × 40 cm plexiglas chamber was set in a quiet laboratory room. The area, 10 cm to the walls, was defined as the wall area. During the test, the mouse was placed in the center of the open-field chamber and allowed to move for 10 min. After each testing trial, the chambers were thoroughly cleaned by 70% ethanol to get rid of odors left by animals tested in a previous trial. The total distance traveled and the time spent in different areas were recorded.

### Data Analysis

Data were presented as the mean ± SEM. For behavioral data and cell counting data, analysis of variance (ANOVA) was conducted to compare main genotype effects. For biochemical results, two-tailed Student’s *t*-test was performed to examine the difference between control and cKO mice; *p* < 0.05 (^∗^) and *p* < 0.01 (^∗∗^) were considered statistically significant and highly significant, respectively.

## Results

### Reduced Size of the Cortex in *PDK1* cKO Mice

**FIGURE 1:**
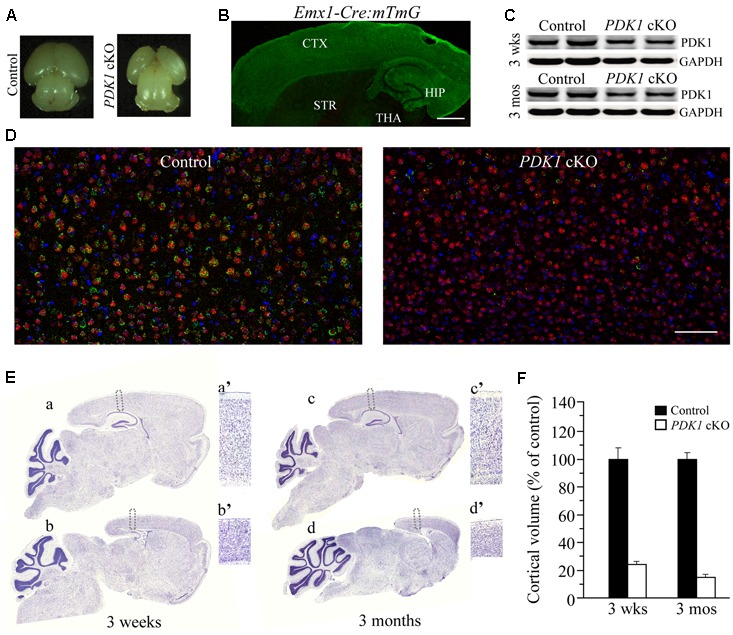
Reduced size of the cortex in forebrain-specific *PDK1* cKO mice. **(A)** Representative brain photos for a control mouse and a *PDK1* cKO mouse at P0. **(B)** The expression pattern of GFP in the brain of a 3-week-old *Emx1-Cre;mTmG* mouse. GFP was mainly expressed in the cortex (CTX) and the hippocampus (HIP) but not the striatum (STR), the thalamus (THA), or the cerebellum. **(C)** Western analysis on PDK1. Cortical homogenates from mice aged at 3 weeks and 3 months (*n* = 3–4/group/age) were prepared. There was significant difference on protein levels of PDK1 between control and *PDK1* cKO mice (3 weeks: control = 100 ± 4.6%, cKO = 58.0 ± 5.8%; 3 months: control = 100 ± 3.5%, cKO = 60.3 ± 2.1%; *p*s < 0.005). GAPDH served as the loading control. **(D)** Double-immunostaining for NeuN/PDK1 in mice at 3 weeks of age. Most NeuN+ cells in control brain were PDK1+ but very few NeuN+ cells in *PDK1* cKO brain were PDK1+. Scale bar = 100 μm. **(E)** Nissl staining for mice at 3 weeks and 3 months. Boxed areas in control (a,c) and cKO (b,d) were enlarged as a’, c’, b’, and d’, respectively. **(F)** Relative cortical volume. There was significant difference on the size of the cortex between control and *PDK1* cKO mice at 3 weeks (control = 100 ± 9.9%, cKO = 24.1 ± 2.0%; *n* = 3–4 mice/group; *p* < 0.001) or 3 months (control = 100 ± 4.4%, cKO = 14.9 ± 1.9%; *n* = 3–4 mice/group; *p* < 0.001).

Early work has shown that *PDK1*^-/-^ mice display multiple abnormalities and die at embryonic day 9.5 (E9.5) (Lawlor et al., 2002). In this study, we have generated viable forebrain-specific *PDK1* cKO (*PDK1^f/f^;Emx1-Cre*) mice. These mutant animals exhibited smaller cerebrum than controls did (**Figure 1A**). To examine the inactivation pattern of PDK1 mediated by Cre recombination, the *mTmG* mouse (Muzumdar et al., 2007) was crossed to the *Emxl-Cre* mouse to obtain *Emxl-Cre;mTmG*. In the latter, the expression of green fluorescence protein (GFP) was mainly observed in the cortex and the hippocampus (**Figure 1B**). To examine the inactivation efficiency of PDK1, we performed Western blotting for PDK1 using cortical homogenates. Significantly reduced PDK1 protein levels were observed in the cortex of *PDK1* cKO mice at 3 weeks and 3 months of age (**Figure 1C**). The residual amount of PDK1 was likely from cells including GABAergic neurons, blood cells, glial cells, and a small proportion of excitatory neurons that do not express the Cre recombinase. We further conducted double-immunostaining for NeuN and PDK1. We found that the majority of cortical NeuN positive (+) cells in *PDK1* cKO mice were PDK1 negative. In contrast, neurons in control mice were doubly positive for NeuN/PDK1 (**Figure 1D**).

Nissl staining was used to examine brain morphology. There was remarkable reduction on the size of the cortex in *PDK1* cKO mice (**Figure 1E**). The thickness of the cortex in *PDK1* cKO mice was decreased by about 50% as compared to age-matched littermate controls (**Figure 1E**a’–d’). Quantification data showed that the cortical volume was dramatically decreased in *PDK1* cKO mice either at 3 weeks or 3 months (**Figure 1F**). In contrast, the size of the cerebellum in *PDK1* cKO mice was not decreased at either age (**Figure 1E**), likely due to that Cre recombinase was not expressed in cerebellar neurons. Moreover, the measurement on the averaged area of the cerebellum per section showed no significant difference between two genotypes at either age (3 weeks: control = 100 ± 2.6%, cKO = 103.1 ± 2.3%; 3 months: control = 100 ± 3.5%, cKO = 99.4 ± 3.7%; *p*s > 0.6, Student’s *t*-test).

### Loss of Mature Neurons in *PDK1* cKO Mice

**FIGURE 2:**
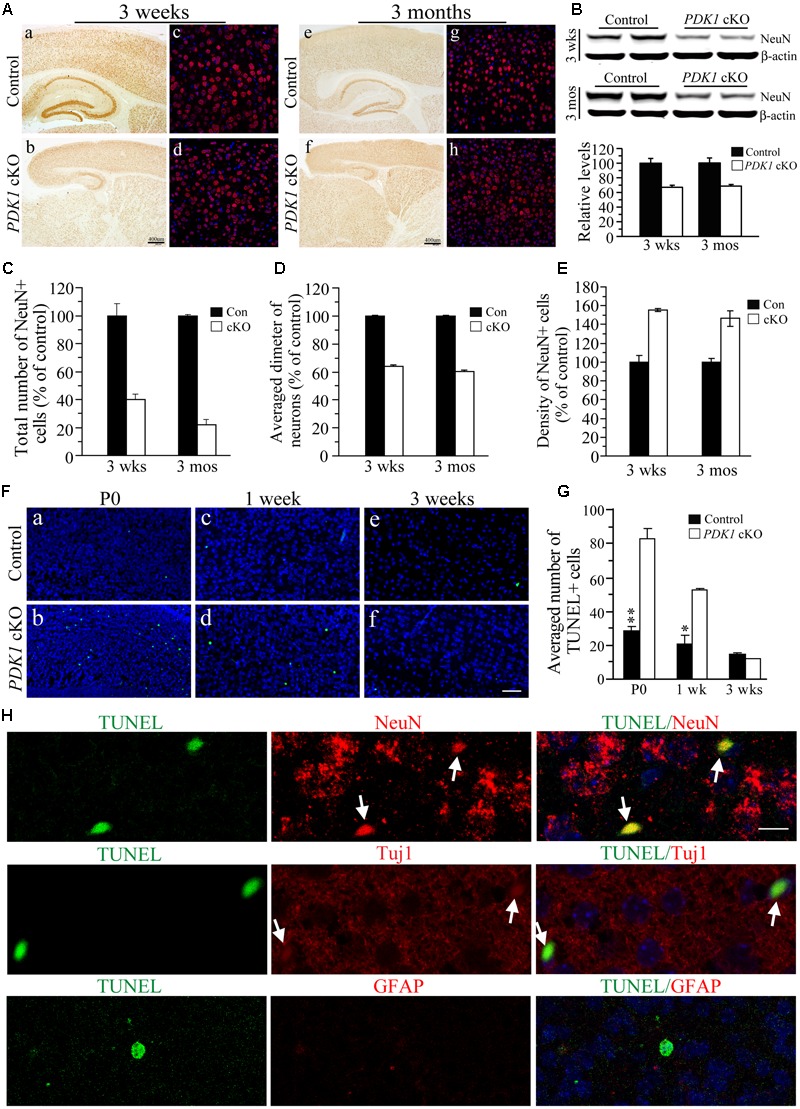
Remarkable loss of mature neurons in *PDK1* cKO mice. **(A)** IHC for NeuN. There were significant reductions on NeuN+ cells in the cortex and the hippocampus of *PDK1* cKO mice at 3 weeks (a–d) and 3 months (e–h). Scale bar = 50 μm. **(B)** Western blotting on NeuN. There were significant differences on NeuN protein levels between control and *PDK1* cKO groups at 3 weeks and 3 months (*p*s < 0.01). β-actin served as the loading control. **(C)** Relative number of NeuN+ cells was significantly different between control and *PDK1* cKO mice at 3 weeks (control = 100 ± 9.8%, cKO = 39.8 ± 3.8%; *n* = 3–4/group; *p* < 0.005) or 3 months (control = 100 ± 0.8%, cKO = 22.0 ± 3.6%; *n* = 3–4/group; *p* < 0.001). **(D)** Relative diameter of NeuN+ cells was significantly decreased in *PDK1* cKO mice at 3 weeks (control = 100 ± 0.2%, *n* = 118 cells from 3 mice; cKO = 60.5 ± 0.1%, *n* = 345 cells from 3 mice; *p* < 0.001) or 3 months (control = 100 ± 0.2%, *n* = 110 cells from 3 mice; cKO = 64.1 ± 0.2%, *n* = 365 cells from 3 mice; *p* < 0.001). **(E)** Cell density of NeuN+ neurons (per 1 mm^3^ area) was significantly increased in *PDK1* cKO mice at 3 weeks (control = 100 ± 3.9%, cKO = 146.3 ± 8.4%; *n* = 4 mice/group; *p* < 0.01) or 3 months (control = 100 ± 7.2%, cKO = 155.5 ± 1.7%, *n* = 4 mice/group; *p* < 0.001). **(F)** TUNEL staining in the cortex of the mice aged at P0 (a,b), 1 week (c,d), and 3 weeks (e,f). Scale bar = 50 μm. **(G)** The averaged number of TUNEL+ cells per section. There were significant increases in *PDK1* cKO mice at P0 and 1 week but not 3 weeks (^∗^*p* < 0.05; ^∗∗^*p* < 0.01). **(H)** Double-immunostaining for TUNEL/NeuN, TUNEL/Tuj1, or TUNEL/GFAP. Cells doubly positive for TUNEL(green)/NeuN(red) or TUNEL(green)/Tuj1(red) were indicated by white arrows in *PDK1* cKO mice. Cells doubly positive for TUNEL(green)/GFAP(red) were not found. Scale bar = 10 μm.

We measured the number of mature neurons using NeuN as a marker. First, IHC on NeuN revealed significant reductions in the cortex size and the total number of NeuN+ cells in *PDK1* cKO mice aged at 3 weeks and 3 months (**Figure 2A**). Consistent with this, Western analysis confirmed reduced NeuN levels (**Figure 2B**). Second, a stereological cell counting method was used to count NeuN+ cell number (West and Gundersen, 1990; Tabuchi et al., 2009; Chen et al., 2010). Our results showed that the total number of NeuN+ cells was dramatically decreased in *PDK1* cKO mice (**Figure 2C**). The measurement on the diameter for the cell body of NeuN+ cells indicated smaller size of neurons in *PDK1* cKO mice than in controls (**Figure 2D**). However, the density of NeuN+ cells in *PDK1* cKO mice was increased as compared to control animals (**Figure 2E**). This finding was in agreement with that reported on *PDK1* conditional knock-in mice (Cordon-Barris et al., 2016). Overall, conditional deletion of PDK1 in the forebrain resulted in remarkable loss of mature neurons.

To investigate whether neuron loss occurred via apoptosis, we conducted the TUNEL assay (Tabuchi et al., 2009). Brain sections of *PDK1* cKO mice aged at P0, 1 week, and 3 weeks were used. We found that the total number of TUNEL+ cells in the cortex of PDK1 mutants was increased (**Figure 2F** and Supplementary Figure 1). Cell counting results confirmed that the averaged number of TUNEL+ cells per section in *PDK1* cKO mice was significantly larger than that in controls (**Figure 2G**: *F* = 60, df1/8, *p* < 0.001 and Supplementary Figure 1), suggesting enhanced apoptotic cell death. To identify which cell type underwent apoptosis, we performed double-staining for TUNEL/NeuN, TUNEL/Tuj1, or TUNEL/GFAP (**Figure 2H**). Only TUNEL+/NeuN+ and TUNEL+/Tuj1+ cells (**Figure 2H**, indicated by white arrows) were observed in *PDK1* cKO mice as compared to controls. No TUNEL+/GFAP+ cells were detected (**Figure 2H**).

### Unchanged Self-renewal of NPCs in *PDK1* cKO Mice

To determine the possibility that loss of cortical neurons was caused by deficient self-renewal of NPCs, we first performed BrdU pulse-labeling experiment, in which BrdU labels NPCs at the S-phase of the cell cycle. No qualitative changes on BrdU immunoreactivity were detected in *PDK1* cKO mice at embryonic day 13.5 (E13.5), E15.5 or E17.5, as compared to age-matched controls (Supplementary Figure 2A). Cell counting results showed no significant reduction in the averaged number of BrdU+ cells per 100 μm × 200 μm area of the VZ/SVZ (sub-VZ) in *PDK1* cKO embryos (Supplementary Figure 2B: *p* > 0.2 for each age).

We next conducted FIHC for PH3, a marker for NPCs at the M-phase of the cell cycle. There was no qualitative difference in the immunoreactivity of PH3 between control and *PDK1* cKO embryos (Supplementary Figure 2C). The averaged number of PH3+ cells in the surface of the VZ for *PDK1* cKO embryos was not significantly decreased (Supplementary Figure 2D: *p* > 0.2 for each age). Overall, the self-renewal or proliferation of NPCs was not impaired in *PDK1* cKO mice.

### Loss of Synapses and Dendrites in *PDK1* cKO Mice

**FIGURE 3:**
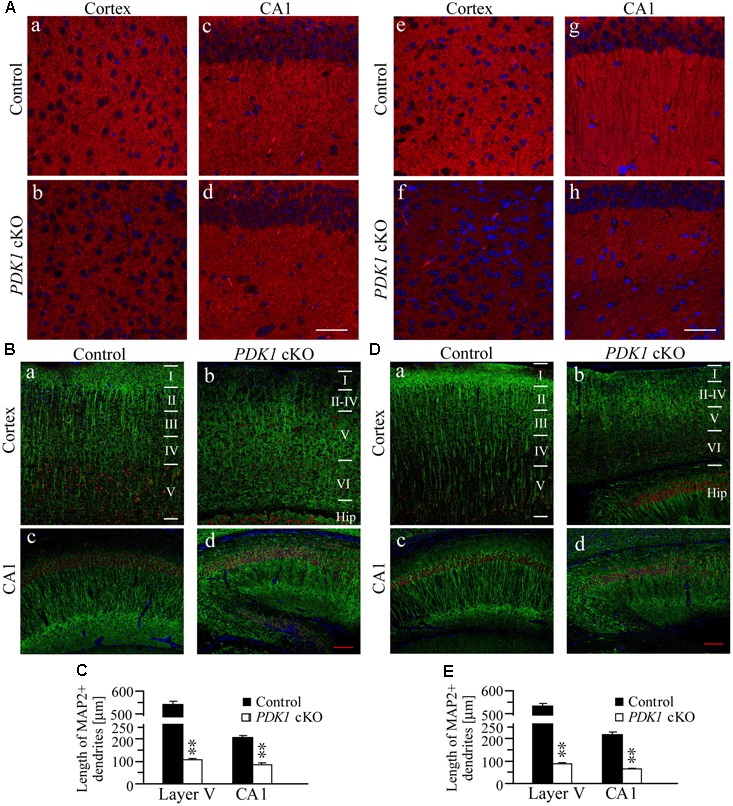
Loss of synapses and dendrites in *PDK1* cKO mice. **(A)** IHC for SVP38. Qualitatively altered immunoreactivity of SVP38 in the cortex and the hippocampus in *PDK1* cKO mice aged at 3 weeks (a–d) or 3 months (e–h). Scale bar = 100 μm. **(B)** Double-staining of MAP2/Ctip2 in the cortex (a,b) and the hippocampus (c,d) of mice at 3 weeks. Dendrites, neurons, and cell bodies were labeled by MAP2 (green), Ctip2 (red), and DAPI (blue), respectively. Scale bar = 100 μm. **(C)** There were significant differences on the averaged dendritic length for neurons in layer V of the cortex and in hippocampal CA1 area of control and *PDK1* cKO mice (30 Ctip2+ neurons from 3 mice per brain area per group; ^∗∗^*p* < 0.01). **(D)** Double-staining of MAP2/Ctip2 in mice at 3 months (a–d). Scale bar = 100 μm. **(E)** The averaged length for dendrites was significantly different between two groups (30 Ctip2+ neurons from 3 mice per brain area per group; ^∗∗^*p* < 0.01).

To study whether the morphology of synapses and dendrites was affected in *PDK1* cKO mice, we first conducted FIHC on synaptophysin (SVP38), a marker for pre-synaptic element. Qualitatively reduced intensity of SVP38 immunoreactivity was observed in the cortex and the hippocampus of *PDK1* cKO mice across ages (**Figure 3A**), suggesting loss of synapses. In addition, Western analyses on SVP38 and post-synaptic density 95 (PSD95) showed that their levels were significantly decreased in *PDK1* cKO mice (data not shown). We then performed double-immunostaining of MAP2/Ctip2, markers for dendrites and pyramidal neurons in cortical layer V, respectively. We found that the immunoreactivity of MAP2 was qualitatively reduced in the cortex of *PDK1* cKO mice as compared to age-matched littermate controls (**Figures 3B,D**), suggesting loss of dendrites. We further measured the length of dendrites of Ctip2+ neurons. The averaged dendritic length was significantly decreased in *PDK1* cKO mice either at 3 weeks (**Figure 3C**) or 3 months (**Figure 3E**). For neurons in cortical layer V, there was more than 80% of reduction on the averaged dendritic length in PDK1 mutants. For pyramidal neurons in hippocampal CA1, the averaged dendritic length was also dramatically reduced in *PDK1* cKO mice (**Figures 3C,E**).

### Astrocytosis in *PDK1* cKO Mice

**FIGURE 4:**
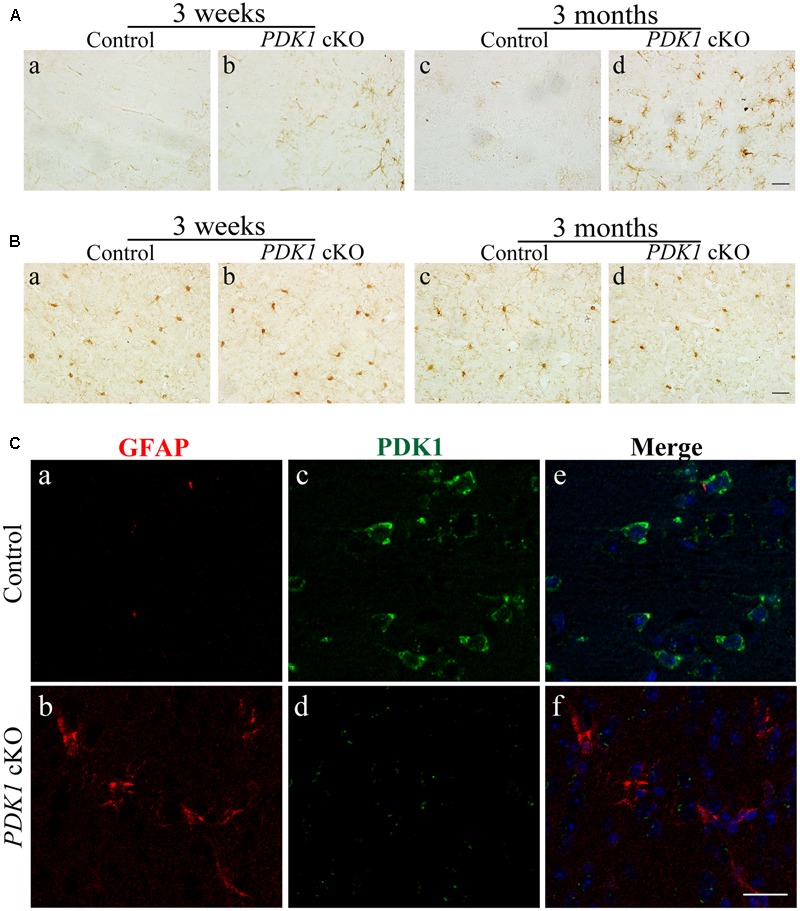
Astroglial activation in *PDK1* cKO mice. **(A)** IHC for GFAP. There was increased number of GFAP+ cells in the cortex of *PDK1* cKO mice at 3 weeks or 3 months. Scale bar = 20 μm. **(B)** IHC for Iba1. There was no significant change on the total number of Iba1+ cells in the cortex of *PDK1* cKO mice at 3 weeks or 3 months. Scale bar = 20 μm. **(C)** Double-immunostaining for GFAP/PDK1. Abundant PDK1+ neurons but few GFAP+ cells were seen in the cortex of control mice (a,c,e). GFAP+ cells were PDK1 negative in *PDK1* cKO mice (b,d,f). Scale bar = 25 μm.

To study whether there were changes on glial cells in the cortex of *PDK1* cKO mice, first, IHC on GFAP was performed. Increased number for GFAP+ cells was observed in the cortex of *PDK1* cKO mice. The increase in the immunoreactivity of GFAP was subtle in *PDK1* cKOs at 3 weeks (**Figure 4A**a,b) but more robust at 3 months (**Figure 4A**c,d), suggesting progressive astroglial activation. Second, IHC on Iba1 was conducted. The immunoreactivity of Iba1 in control and *PDK1* cKO mice did not qualitatively differ at either age (**Figure 4B**), indicating no significant microgliosis.

Since the Cre recombinase was also expressed in astrocytes in *PDK1^f/f^;Cre* mice, this suggested a possibility that loss of PDK1 may affect the cell number of astrocytes. We conducted double-immunostaining for GFAP/PDK1. We found that GFAP+ cells were largely PDK1 negative in *PDK1* cKO mice (**Figure 4C**), indicating that PDK1 was inactivated in astrocytes. Moreover, it has been demonstrated that astrocytosis is associated with neuron loss in neurodegenerative mouse models (Saura et al., 2004; Tabuchi et al., 2009; Cheng et al., 2015). Overall, astroglial activation in *PDK1* cKO mice may be due to neuronal death and a cell autonomous function of PDK1 in astrocytes.

### Learning Deficit in *PDK1* cKO Mice

**FIGURE 5:**
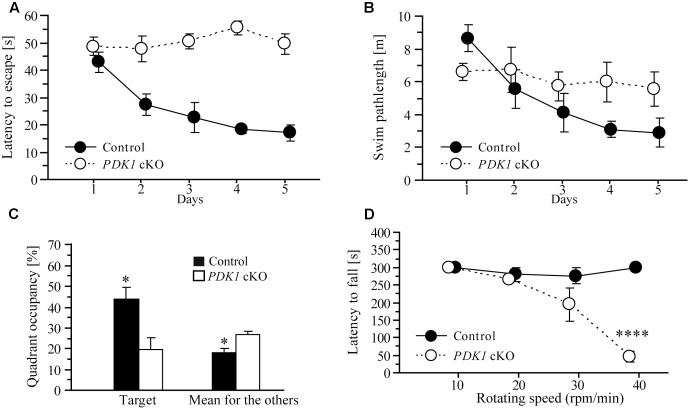
Learning deficit in *PDK1* cKO mice. **(A)** Escape latency for control and *PDK1* cKO mice which were trained with a hidden platform task for 5 consecutive days. There was significant difference on the latency to escape between the two genotype groups (*n* = 6/group). *PDK1* cKO mice showed no improvement on their performance during the 5-day training. **(B)** The length of swim-path. There was significant difference between control and *PDK1* cKO mice across the training period. **(C)** Quadrant occupancy in a probe test conducted 24 h after the last training trial. There was significant difference on the time spent in the target quadrant between control and *PDK1* cKO mice (^∗^*p* < 0.05). There was also significant difference in the average time spent in the remaining three quadrants (adjacent left, adjacent right, and opposite) between control and *PDK1* cKO animals (^∗^*p* < 0.05). **(D)** Latency to fall from a rotarod. Four different rotating speeds including 10, 20, 30, and 40 rpm/min were used. There was no significant difference on the latency to fall between control (*n* = 10) and *PDK1* cKO mice (*n* = 6) at lower speeds (*p*s > 0.1). There was significant difference between control and *PDK1* cKO mice at the highest speed (^∗∗∗∗^*p* < 0.001).

To determine whether cognitive ability of PDK1 mutant mice was affected, we used a Morris water maze task to test spatial learning. In this task, 2- to 3-month-old mice were trained to learn a hidden platform for 5 days. During the 5-day training period, there was no improvement on the latency to escape in *PDK1* cKO mice. ANOVA confirmed a highly significant main genotype effect (*F* = 46.1, *df* = 1/10, *p* < 0.001) between two groups of mice across 5 days (**Figure 5A**). There was also significant genotype effect (*F* = 11.6, *df* = 3.0/30.1, *p* < 0.001) on the length of swim-path (**Figure 5B**). Overall, these results indicated that spatial learning was severely affected in *PDK1* cKO mice.

One day after the last training trial, the mice were subjected to a probe test, in which no platform was available in the water. The average time spent in the target and other quadrants of the water maze was analyzed (**Figure 5C**). Significant genotype effect (*F* = 8.5, *df* = 1/10, *p* < 0.05) and significant quadrant × genotype effect (*F* = 4.0, *df* = 2.4/23.9, *p* < 0.05, Greenhouse-Geisser correction) were observed, suggesting that no spatial memory had formed in *PDK1* cKO mice. We also found that the control mouse preferred to search for the target quadrant but the *PDK1* cKO swam randomly during the probe test (data not shown).

Next, a rotarod task was conducted to examine motor learning. A number of different rotating speeds including 10, 20, 30, and 40 rpms/min were used. The latency to fall from the rotating rod was averaged (**Figure 5D**). ANOVA revealed a significant speed × genotype effect (*F* = 22.8, *df* = 1.5/21.0, *p* < 0.001). At lower speeds including 10, 20, or 30 rpm/min, there were no significant genotype effects on the latency to fall between control and *PDK1* cKO mice (*p*s > 0.1). However, there was significant genotype effect at the speed of 40 rpm/min (*p* < 0.001), likely suggesting impairment on motor learning.

### Decreased Activities for Akt and mTORC1 in *PDK1* cKO Mice

**FIGURE 6:**
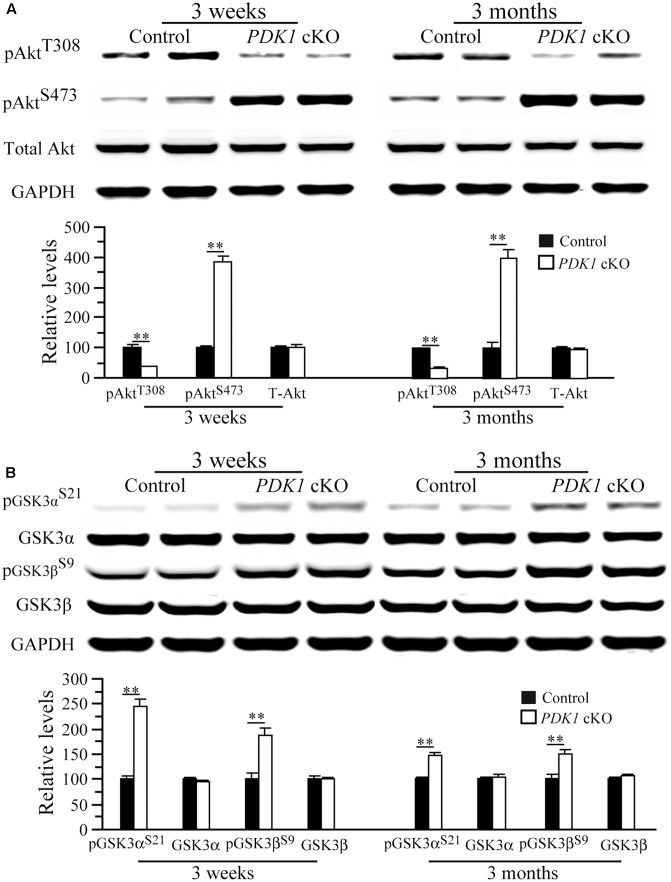
Levels of pAkt and pGSK3 in *PDK1* cKO mice. **(A)** Western analyses on pAkt and total Akt (T-Akt). There were significant differences on relative levels of pAkt^T308^ and pAkt^S473^ between control and *PDK1* cKO mice at 3 weeks and 3 months (^∗∗^*p* < 0.01; *n* = 3–4/group/age). There was no significant difference on T-Akt levels between control and *PDK1* cKO mice at either age (*p*s > 0.2). GAPDH served as the loading control. **(B)** Western analyses on pGSK3α, pGSK3β, total GSK3α, and total GSK3β. There were significant differences on relative levels of pGSK3α^S21^ and pGSK3β^S9^ between control and *PDK1* cKO mice at 3 weeks and 3 months (^∗∗^*p* < 0.01). There were no significant differences on levels of total GSK3α and GSK3β between control and *PDK1* cKO mice (*p*s > 0.5). GAPDH served as the loading control.

Our biochemical analysis showed that levels of total Akt (T-Akt) were not changed in *PDK1* cKO mice at either age (**Figure 6A**, *p*s > 0.2), indicating unaltered Akt expression. In contrast, relative levels of pAkt^Thr308^ were decreased in *PDK1* cKO mice (**Figure 6A**) (3 weeks: control = 100 ± 10.5%, cKO = 37.0 ± 1.1%; 3 months: control = 100 ± 1.2%, cKO = 35.5 ± 1.3%; *n* = 3–4/group/age; *p*s < 0.01). In contrast, levels of pAkt^Ser473^ were increased in *PDK1* cKO mice (**Figure 6A**, *p*s < 0.01). In agreement with this, increased levels of pAkt^Ser473^ were reported in the brain of *PDK1^f/f^;GFAP-Cre* mice (Figure 4 in Chalhoub et al., 2009).

The finding of reduced pAkt^Thr308^ levels suggested that Akt activity might be decreased. As a major Akt substrate, GSK3 was then examined. Levels of total GSK3α and GSK3β did not differ between two groups (**Figure 6B**), but those for pGSK3α^Ser21^ and pGSK3β^Ser9^ were increased in *PDK1* cKO mice either at 3 weeks (pGSK3α: control = 100 ± 6.3%, cKO = 244.8 ± 15.1%; pGSK3β: control = 100 ± 11.2%, cKO = 187.3 ± 13.7%; *p*s < 0.01) or 3 months (pGSK3α: control = 100 ± 1.6%, cKO = 146.6 ± 5.3%; pGSK3β: control = 100 ± 8.0%, cKO = 148.5 ± 7.4%; *p*s < 0.01; *n* = 3–4/group/age). Due to negative correlation between pGSK3α^Ser21^/3β^Ser9^ levels and GSK3 activity, this finding suggested that GSK3 was inactivated. In agreement with this, increased pGSK3α^Ser21^/3β^Ser9^ was reported in *PDK1^f/f^;GFAP-Cre* mice as well (Figure 4 in Chalhoub et al., 2009).

**FIGURE 7:**
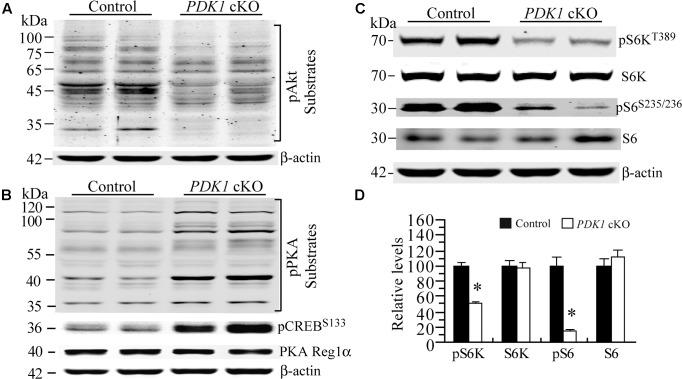
Enhanced PKA activity but decreased mTORC1 activity in *PDK1* cKO mice. **(A)** Western blotting for pAkt substrates. Several bands for Akt downstream targets were decreased in *PDK1* cKO mice (*n* = 3 mice/group). **(B)** Western blotting for pPKA substrates, pCREB and PKA Reg1α. Several PKA downstream targets exhibited increased levels in *PDK1* cKO mice. Levels for pCREB^Ser133^ but not PKA Reg1α were increased (*n* = 3 mice/group). **(C)** Western blotting for pS6K and pS6 in control and *PDK1* cKO mice. **(D)** There were significant differences on relative levels of pS6K and pS6 between control and *PDK1* cKO mice at 3 months (*n* = 3 mice/group; ^∗^*p* < 0.05). There were no significant differences on levels of total S6K and S6 between control and *PDK1* cKO mice (*p*s > 0.5). β-actin was used as the loading control.

It has been shown that GSK3 is phosphorylated by several kinases including Akt (Cross et al., 1995) and PKA (Fang et al., 2000; Li et al., 2000). First, Western analysis on pAkt substrates was performed to examine overall Akt activity. Our results showed that levels for several bands of Akt substrates were reduced in *PDK1* cKO mice as compared to controls (**Figure 7A**), suggesting decreased activity. Second, Western analysis on pPKA substrates was performed. A number of pPKA substrates exhibited increased levels (**Figure 7B**), suggesting enhanced activity. Consistent with this, relative levels for pCREB^Ser133^, a well-known PKA substrate, were increased in *PDK1* cKO mice (**Figure 7B**: control = 100 ± 5.8%; cKO = 387.1 ± 37.2%; *p* < 0.01). In contrast, levels of PKA regulatory subunit 1α (PKA Reg1α) were not changed (**Figure 7B**: control = 100 ± 1.2%; cKO = 99.1 ± 7.2%; *p* > 0.9).

Previous evidence has demonstrated that the mTOR signaling is critical for neuronal survival (Sarbassov et al., 2005; Kim et al., 2010) and dendritic morphology (Jaworski et al., 2005; Kumar et al., 2005). To investigate whether mTORC1 activity was affected, we examined pS6K and pS6 using cortical samples at 3 months. We found that levels for total S6K and S6 were not changed in *PDK1* cKO mice (**Figures 7C,D**, *p*s > 0.5). However, those for pS6K^Thr389^ and pS6^Ser235/236^ were dramatically decreased (*p*s < 0.05). Overall, mTORC1 activity was decreased in *PDK1* cKO mice.

## Discussion

PDK1 is a key member in the PI3K signaling (Mora et al., 2004; Engelman et al., 2006) and has been implicated in neurological diseases (Liu et al., 2011; Pietri et al., 2013). Early embryonic lethality of *PDK1^-/-^* mice excludes the possibility to study whether loss of endogenous PDK1 affects neuronal survival during cortical development. In this study, viable forebrain-specific *PDK1* cKO mice were generated. The following novel findings were reported. First, conditional deletion of PDK1 in the forebrain causes dramatic neuron loss and increased apoptotic cell death. Second, conditional deletion of PDK1 in the forebrain results in impaired mTOR activity.

Microcephaly has been observed in early work using *PDK1* cKO line in which PDK1 is conditionally inactivated in neurons and astrocytes of the whole brain (Chalhoub et al., 2009). Given that PDK1 is specifically deleted in the forebrain of *PDK1^f/f^;Emx1-Cre* mice, our findings are somehow consistent with those reported previously (Chalhoub et al., 2009; Itoh et al., 2016). First, the current model differs from the line Chalhoub et al. (2009), in that the phenotype in the cerebellum is not the same. This is likely due to that the inactivation of PDK1 does not occur in neurons of the cerebellum in our cKO line. Second, unlike *PDK1^f/f^;GFAP-Cre* (Chalhoub et al., 2009) or *PDK1^f/f^;Emx1-Cre* (this study), *PDK1^f/f^;Nestin-Cre* mice die shortly after birth (Itoh et al., 2016) and therefore cannot be used for the study on postnatal brain. Third, *PDK1^f/f^;Nex-Cre* mice survive to adulthood and display cortical lamination defect (Itoh et al., 2016). Moreover, it has been nicely demonstrated that abnormal cortical lamination in *PDK1^f/f^;Nex-Cre* mice is caused by impairment on PDK1/Akt-dependent neuronal migration (Itoh et al., 2016).

Since the total number of NeuN+ cells is dramatically reduced and the length of dendrites is significantly decreased in *PDK1* cKO mice, these could directly lead to the formation of small cortex. Overall, endogenous PDK1 may negatively regulate apoptosis in the cortex and therefore control the brain/cortex size. Interestingly, apoptosis is involved in cell death in brain diseases displaying age-related neuron loss. First, previous studies have shown that increased apoptosis is associated with neuron loss in neurodegenerative mouse models (Feng et al., 2004; Tabuchi et al., 2009; Wines-Samuelson et al., 2010; Cheng et al., 2015). Second, early work has demonstrated increased apoptotic cell death in the brain of AD (Su et al., 1994; Lassmann et al., 1995; Anderson et al., 1996).

Since increased number of TUNEL+/NeuN+ or TUNEL+/Tuj1+ cells was found in *PDK1* cKO mice, it was reasonable to conclude that neuronal apoptosis contributes to cortical neuron loss. In contrast, neuron loss in *PDK1* cKO mice is unlikely due to proliferation of NPCs during development, since our results showed that the self-renewal of NPCs was not impaired as revealed by the BrdU pulse-labeling and PH3 IHC experiments.

To explore the underlying molecular mechanisms, we focused on the Akt/mTOR pathway. First of all, our biochemical results on pAkt^Thr308^ and pAkt substrates have strongly suggested that conditional inactivation of PDK1 leads to reduced Akt activity. Second, our analyses on pS6K and pS6 indicated decreased activity of mTORC1 in *PDK1* cKO mice. Third, *PDK1^f/f^;Emx1-Cre* mice exhibit increased pGSK3 levels, which may be caused by enhanced PKA activity. Consistent with this notion, previous evidence has shown that PKA inhibits GSK3 by phosphorylating the Ser21/Ser9 of GSK3α/3β (Fang et al., 2000), and that the phosphorylation of GSK3 by PKA does not require activation of Akt (Li et al., 2000). Overall, since PDK1, Akt, or mTOR is important for cell/neuron survival and dendritic morphogenesis (Datta et al., 1997; Jaworski et al., 2005; Kumar et al., 2005; Sarbassov et al., 2005; Kharebava et al., 2008; Kim et al., 2010), it is reasonable to conclude that PDK1 may control neuronal survival during cortical development via the activation of Akt/mTOR signaling.

In this study, we report that *PDK1* cKO mice display deficit on spatial learning and memory. Since it has been demonstrated that neuron loss and synaptic loss directly contribute to cognitive impairments in neurodegenerative disease (Gomez-Isla et al., 1997; Terry, 2000), we reason that learning deficit in *PDK1* cKO mice is likely caused by massive loss of neurons and synapses in the cortex. However, since there was an increase in open-field activity in *PDK1* cKO mice, it cannot be ruled out that increased anxiety may directly cause learning impairment or exacerbate learning deficit in PDK1 mutant mice.

## Author Contributions

CX, LY, JH, RJ, HW, CH, TL, QW, and XZ conducted experiments. GC, YX, ZYY, and ZZY designed experiments. GC, ZYY, CX, and LY analyzed the data. GC, TS-J, and RM wrote the article.

## Conflict of Interest Statement

The authors declare that the research was conducted in the absence of any commercial or financial relationships that could be construed as a potential conflict of interest.

## Footnotes

1 https://imagej.nih.gov/ij/
