# Supplementary figures and images for: Conditional Deletion of PDK1 in the Forebrain Causes Neuron Loss and Increased Apoptosis during Cortical Development
Source: Front Cell Neurosci. 2017 Oct 20;11:330. doi: 10.3389/fncel.2017.00330 (PMC5655024; doi:10.3389/fncel.2017.00330)

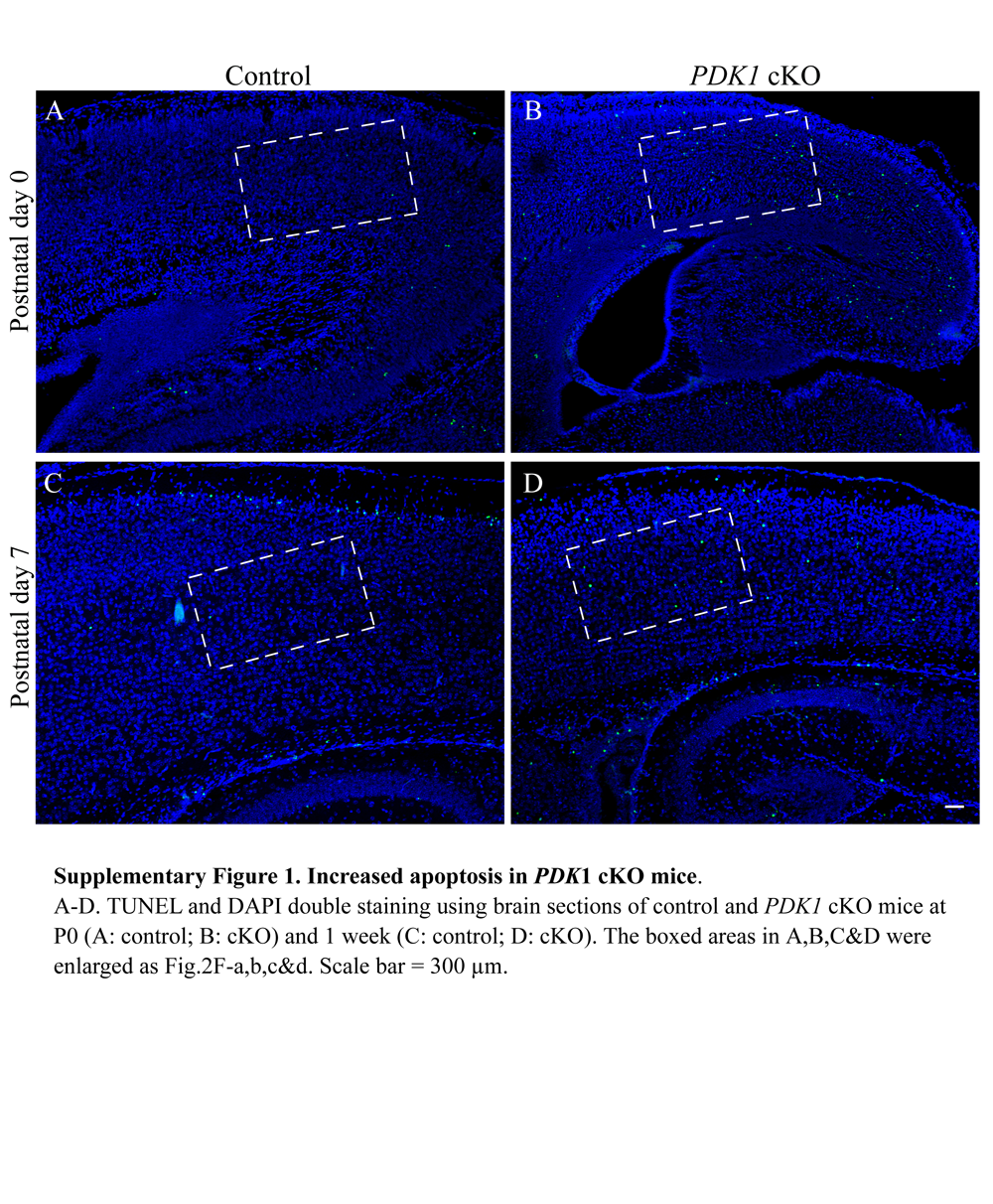

Supplement: Supplementary file 1 [file Image_1.TIF]

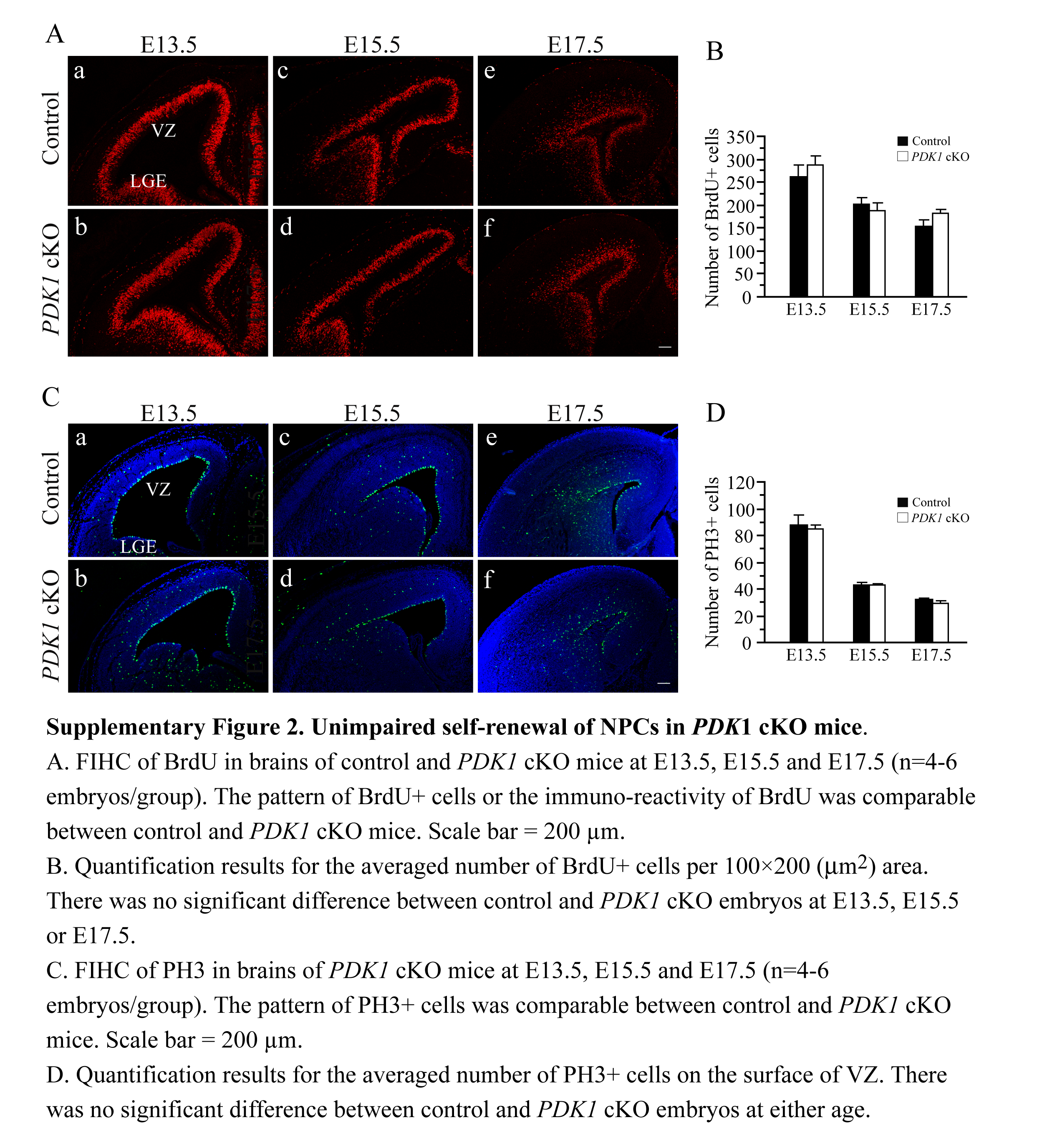

Supplement: Supplementary file 2 [file Image_2.TIF]
